# Pax-5 is a potent regulator of E-cadherin and breast cancer malignant processes

**DOI:** 10.18632/oncotarget.14511

**Published:** 2017-01-05

**Authors:** Sami Benzina, Annie-Pier Beauregard, Roxann Guerrette, Stéphanie Jean, Mame Daro Faye, Mark Laflamme, Emmanuel Maïcas, Nicolas Crapoulet, Rodney J. Ouellette, Gilles A. Robichaud

**Affiliations:** ^1^ Université de Moncton, Département de chimie et biochimie, Moncton, NB, E1A 3E9, Canada; ^2^ Atlantic Cancer Research Institute, Moncton, NB, E1C 8×3, Canada; ^3^ Department of Fisheries and Oceans Canada, Molecular Biology Unit, Moncton, NB, E1C 9B6, Canada; ^4^ Georges-L.-Dumont University Hospital Centre, Pathology Department, Moncton, NB, E1C 2Z3, Canada

**Keywords:** Pax-5, breast cancer, EMT-MET, E-cadherin, metastasis

## Abstract

Pax-5, an essential transcription factor for B lymphocyte development, has been linked with the development and progression of lymphoid cancers and carcinoma. In contrast to B-cell cancer lesions, the specific expression signatures and roles of Pax-5 in breast cancer progression are relatively unknown. In the present study, we set out to profile Pax-5 expression in mammary tissues and elucidate the cellular and molecular roles of Pax-5 in breast cancer processes. Using immunohistology on mammary tissue arrays, Pax-5 was detected in a total of 298/306 (97.6%) samples tested. Interestingly, our studies reveal that Pax-5 inhibits aggressive features and confers anti-proliferative effects in breast carcinoma cells in contrast to its oncogenic properties in B cell cancers. More precisely, Pax-5 suppressed breast cancer cell migration, invasion and tumor spheroid formation while concomitantly promoting cell adhesion properties. We also observed that Pax-5 inhibited and reversed breast cancer epithelial to mesenchymal phenotypic transitioning. Mechanistically, we found that the Pax-5 transcription factor binds and induces gene expression of E-cadherin, a pivotal regulator of epithelialisation. Globally, we demonstrate that Pax-5 is predominant expressed factor in mammary epithelial cells. We also present an important role for Pax-5 in the phenotypic transitioning processes and aggressive features associated with breast cancer malignancy and disease progression.

## INTRODUCTION

Breast cancer is still one of the most prevalent types of cancer diagnosed in women where metastasis accounts for 90% of deaths. Although the mechanisms driving breast cancer metastasis are not completely understood, there is a general agreement that the metastatic cascade encompasses alterations in phenotypic features which grant mammary epithelial cells the capacity to invade other tissues and establish metastatic tumors. These processes are also known as: the epithelial-mesenchymal transition (EMT) where tumor cells gain invasive properties to spread into the circulatory system and distant organs and; the mesenchymal-epithelial transition (MET) where metastatic cancer cells reboot an epithelial program to colonize and develop metastatic niches. Currently, there is a poor understanding of the mechanisms coordinating EMT and MET programs and how they are regulated in cancer progression.

One pivotal feature in the metastatic cascade is the loss of the epithelial cell adhesion molecule E-cadherin [1, 2]. In fact, not only is E-cadherin a predominant surface epithelial phenotype marker, but also, a pivotal regulator of anti-invasive properties and MET [3–5]. Consequently, loss of E-cadherin expression not only disrupts cell–cell junctions; but also, correlates with the acquisition of invasiveness, increased tumor grade *in vitro* and *in vivo* [6, 7], and poor patient prognosis [8, 9]. Mechanistically, membrane-bound E-cadherin prevents nuclear signaling and transcriptional activation of mesenchymal genes, EMT and cancer progression [2, 10, 11]. Studies have also identified multiple negative regulators for E-cadherin expression such as: Snail [12], Twist [13], Slug [14], and ZEB [15] which are deployed in various carcinomas during phenotypic transitioning and disease progression.

Recently, we and others have suggested a role for *Pax-5* in phenotypic transitioning programs (EMT-MET) which in turn could modulate breast cancer aggressivity and disease progression [16–18]. *Pax-5* is a member of the Paired Box (*Pax*) gene family that controls gene expression programs pivotal in cellular processes such as proliferation, differentiation and apoptosis [19]. Pax-5 has been extensively studied for its essential role in B lymphocyte identity and commitment [20]. Functional studies have shown that *Pax-5* behaves as a potent oncogene in most types of lymphoma and lymphocytic leukemia [21]. We now know that *Pax-5* expression is found in a variety of cell types and non-lymphoid cancers such as: neuroblastoma, rhabdomyosarcoma, merkel- and small-cell carcinomas, oral carcinomas, colorectal carcinoma, neuroendocrine carcinoma, bladder carcinoma, lung carcinoma, liver carcinoma (reviewed in [22]). Although controversial, *Pax-5* expression has also been detected in breast carcinoma [23–25]. Intriguingly, *Pax-5* seems to confer an anti-proliferative effect in most carcinomas studied in opposition to its oncogenic effects in B cell cancers [18, 26]. In contrast to B-cell cancer lesions, the specific role of *Pax-5* in carcinoma development and progression is relatively unknown.

In the present study, we characterize *Pax-5* expression profiles in breast cancer using mammary tissue-arrays and show that *Pax-5* expression is prevalent in 97% of mammary samples tested. We also elucidate the molecular and cellular roles of *Pax-5* in breast cancer processes. More importantly, we show that *Pax-5* is a potent inducer of pro-epithelialisation regulator E-cadherin which leads to breast cancer MET. These findings bring a better understanding of the genetic triggers and signaling networks regulating breast cancer malignancy which is essential for a comprehensive understanding of disease progression and to improve patient outcome.

## RESULTS

### Pax-5 is expressed in mammary cell lines

**Figure 1:**
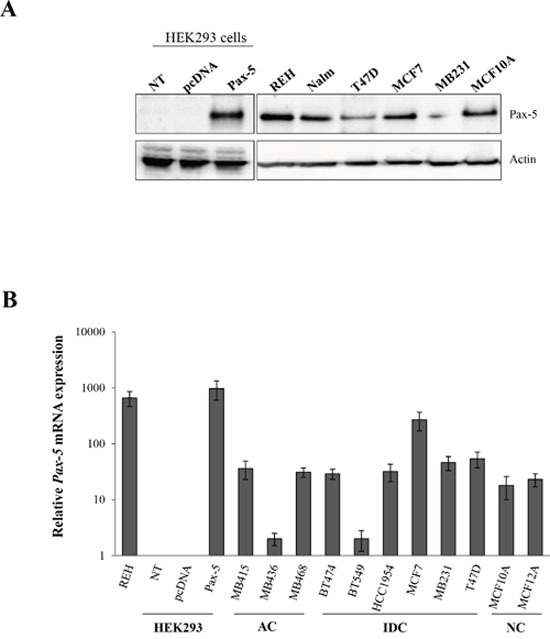
Relative *Pax-5* expression in breast cancer cell lines *Pax-5* gene expression was assessed in a variety of commonly used breast cancer cell lines. **A**. Western blots were performed on total cell lysates and immuno-probed with anti-Pax-5 or anti-actin antibodies. **B**. Relative *Pax-5* transcriptional expression levels were determined by RT-qPCR and standardized against the HPRT housekeeping gene in each respective cell model. Control samples include *Pax-5* bearing B lymphocytes (Reh and Nalm6) and *Pax-5* negative cells (HEK293) which were either non-transfected (NT) or transfected with recombinant *Pax-5* or the empty vector alone (pcDNA). AC indicates adenocarcinoma; IDC, invasive ductal carcinoma; and NC, non-cancerous. Standard deviations are representative of triplicates from three distinct biological samples.

Recent studies have presented opposing findings pertaining to the putative expression of the *Pax-5* gene in breast carcinoma [18, 27]. We thus set out to profile *Pax-5* gene expression in various mammary cancer cell lines and clinical samples. First, we studied commonly used mammary cell models to determine endogenous Pax-5 protein expression using Western blotting. We observed that the Pax-5α (hereafter called Pax-5) protein is expressed in all cancerous (T47D, MCF7 and MB231) and non-cancerous (MCF10A) breast cell lines tested when compared to Pax-5 positive B cells (REH and Nalm-6) and negative embryonic kidney (HEK293) control cell samples (Figure 1A). To gain a better perspective on *Pax-5* transcript expression profiles from breast cancer cell lines, a collection of commonly used cell models from adenocarcinoma (i.e. MB415, MB436, and MB468), invasive ductal carcinoma (i.e. BT474, BT549, HCC1954, MCF7, MB231 and T47D) and non-cancerous (i.e. MCF10A and MCF12A) mammary cells were assessed for *Pax-5* expression using RT-qPCR (Supplementary Table 1) [28]. We found that all breast cell lines were positive for *Pax-5* mRNA expression when compared to positive (REH) and negative (HEK293) controls (Figure 1B). Generally, we observed that endogenous *Pax-5* transcripts levels were much lower in mammary cells in comparison to B lymphocytes.

### Pax-5 is expressed in primary breast cancer tissues

**Figure 2:**
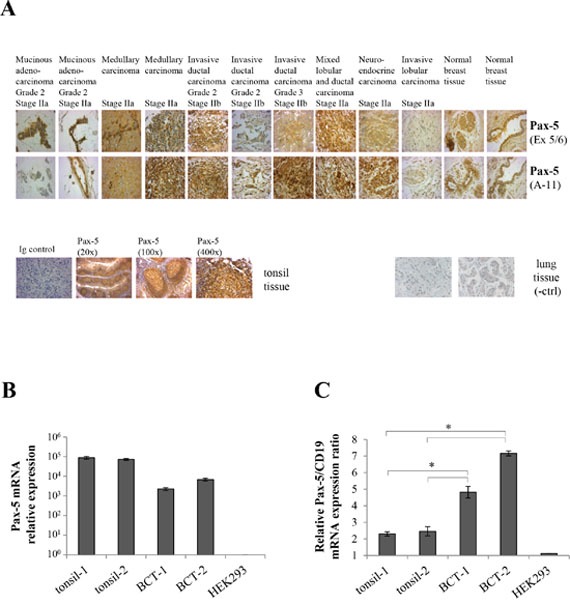
Pax-5 is expressed in clinical breast cancer tissues **A**. Immunohistochemistry was performed on FFPE breast tissue microarrays representing a panel of 306 core samples from healthy and varying mammary cancer types. Tissues slides were probed with anti-Pax-5 antibodies targeting the exon 5/6 (Ex 5/6) and N-terminal (A-11) regions respectively and subsequently revealed with HRP-conjugated host antibodies and hematoxylin counterstaining. Images were taken at a 400X magnification and are representative of duplicate samples from tissues cores of 1.5 um in diameter. Control samples include mammary tissues probed with an isotype-matched irrelevant primary antibody (Ig control); tonsil tissues (*Pax-5*-bearing B cell) used as a positive control; and, cancerous lung tissue as a negative control (-ctrl). Relative mRNA expression levels of *Pax-5* **B**. and *Pax-5/CD19* ratios **C**. were determined by RT-qPCR in clinical FFPE tonsil samples (tonsil-1 and -2) and breast cancer tissues (BCT-1 and BCT-2). HEK293 cells were used as a negative control. The presented data is the calculated mean of three independent samples where statistical analysis by *t*-test indicates significant differences in respect to control cells (* *p*<0.01).

To demonstrate the clinical relevance of our findings, we investigated *Pax-5* expression profiles on formalin-fixed paraffin-embedded (FFPE) tissues arrays from breast cancer patients using immunohistochemistry (IHC) staining. We studied a total of 306 mammary samples using two distinct Pax-5 antibodies targeting the central (exons 5/6) and C-terminal (exons 9/10) regions respectively (Figure 2A). A scoring system was created based on the observed intensities of Pax-5 expression (*i.e*. score of 0 = no expression; 1 = weak; 2 = moderate, and 3 = strong) (Supplementary Table 2). Interestingly, the majority of breast samples tested positive (97%) for Pax-5 protein expression when compared to control samples consisting of positive (B cells from tonsil sections) and negative (lung cancer sections). Globally, Pax-5 expression was common to all breast tissue types (both normal and malignant) with the majority of them (58%) expressing Pax-5 at moderate levels (score 2). Surprisingly, only 8/306 (2.6%) samples tested negative (score 0) for Pax-5 expression. More specifically, we observed Pax-5 expression in: 93% in normal breast; 100% in all invasive ductal and lobular carcinoma; 86% in mucinous adenocarcinoma; and, 100% of ductal carcinoma *in situ* (Supplementary Table 2).

To confirm Pax-5 expression from FFPE samples, we extracted total RNA from two FFPE breast cancer tissue samples (BCT-1 and BCT-2) and performed RT-qPCR to detect *Pax-5* mRNA (Figure 2B). Both cancer tissues were positive for *Pax-5* expression when compared to positive controls (B cells from FFPE tonsil samples). To ascertain that the observed *Pax-5* mRNA results were not due to embedded circulating B lymphocytes, we calculated an expression ratio of *Pax-5* transcripts in relation to the B cell-specific phenotype marker CD19. We found that the *Pax-5/CD19* mRNA expression ratios were up to 3 fold higher in breast cancer tissues in comparison to tonsil tissues (Figure 2C). Together, these results demonstrate that *Pax-5* gene products are commonly expressed in cancerous as well as non-cancerous human breast cells.

In order to draw specific correlations between Pax-5 expression patterns and patient sample physiognomies, we attempted to associate Pax-5 expression profiles to breast cancer pathology subtypes and estrogen receptor (ER) status. Using Spearman algorithms we were unable to demonstrate a correlation between Pax-5 expression and cancer pathology subtypes (data not shown). However, analysis of the scoring data using a two tailed t-test and Pearson rank-order, we revealed a statistically significant correlation between Pax-5 and ER expression in these tissues (with *P* values of 0.029 for ERα and 0.004 for ERβ); although with weak Pearson correlation coefficient of 0.125 and 0.166 respectively for ERα and ERβ. These findings suggest that Pax-5 expression is not specific to a type of breast cancer; however, Pax-5 appears to be co-expressed with ER status in breast cancer cells.

### Pax-5 attenuates breast cancer growth

**Figure 3:**
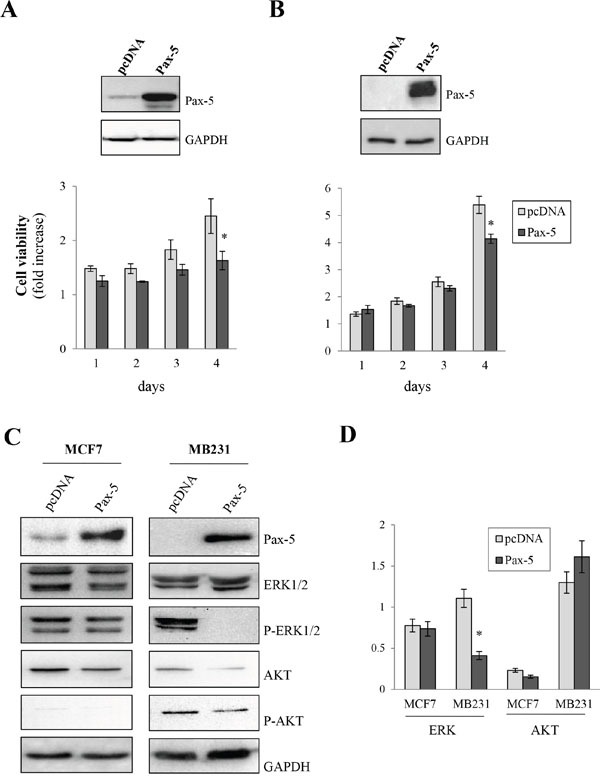
*Pax-5* attenuates breast cancer cell growth MCF7 **A**. and MB231 **B**. cells were transfected with either recombinant *Pax-5* or the empty vector (pcDNA) and monitored for cell growth relative to parental cells over time (1 to 4 days) using a florescent viability assay (CellTiter-Blue, Promega). GAPDH and recombinant *Pax-5* expression were confirmed by Western blots (top panels) for the respective breast cancer models. **C**. Using Western blot, MCF7 and MB231 cells transfected with *Pax-5* or pcDNA were evaluated for the expression and phosphorylation (P) profiles of extracellular-regulated kinases 1/2 (ERK1/2) and AKT (protein kinase B). Immunoblotting of Pax-5 and GAPDH were also performed as control samples. **D**. Results were validated using blot density quantification with pixel density values from phosphorylated forms of ERK and AKT in relation to total ERK and AKT expression levels respectively. The presented data is the calculated mean of three independent samples and is representative of three different experiments (* *p*<0.01).

Given the paucity of data relating to *Pax-5* gene expression in breast cancer cells, we studied the roles of *Pax-5* in breast cancer processes. We first measured cell proliferation rates in MCF7 and MB231 breast cancer cell lines in response to *Pax-5* recombinant expression. Interestingly, *Pax-5* transfections in MCF7 (Figure 3A) and MB231 (Figure 3B) cells attenuated cell viability rates by 34% and 24% respectively at day 4 post-transfection in comparison to vector transfected controls. To determine whether Pax-5-mediated suppression of breast cancer viability was caused by caspase-dependent apoptosis, we monitored caspases 3/7 activity in these cells over time. The relative caspase activity remained unchanged throughout our studies thus suggesting that *Pax-5*-induced suppression of cellular growth is not a result of caspase-dependent apoptosis (data not shown).

To gain a perspective on *Pax-5*-mediated signaling in cellular proliferation, we investigated commonly known pathways regulating cell viability/growth processes. Using Western blot on Pax-5-transfected MCF7 and MB231 cells, we evaluated the expression and phosphorylation levels of extracellular-regulated kinases 1/2 (ERK1/2) and AKT (protein kinase B), two serine/threonine-specific kinases involved in cellular growth signaling. We found that *Pax-5* did not appear to considerably affect total ERK1/2 expression levels (Figure 3C). However, *Pax-5* completely inhibited ERK 1/2 phosphorylation in mesenchymal-dominant MB231 cells. On the other hand, Pax-5 did not appear to modulate the ratio of phosphorylated AKT in relation to total AKT expression (Figure 3D).

### Pax-5 promotes breast cancer cell adherent properties

**Figure 4:**
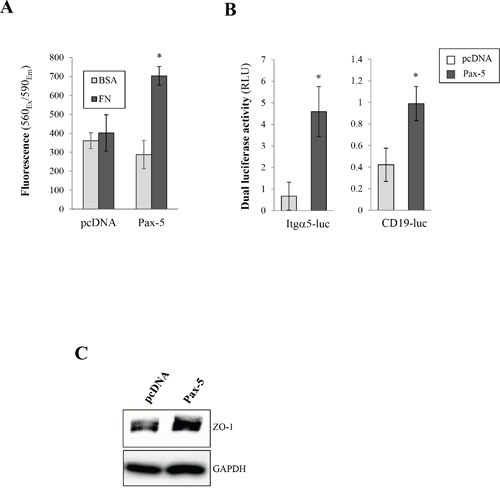
Pax-5 increases breast cancer cell adherent properties **A**. MCF7 cells were transfected with either Pax-5 or the empty vector (pcDNA) and incubated in 96 well microplates pre-coated fibronectin (FN) or bovine serum albumin (BSA); washed; and then analysed using the CellTiter-Blue method (Promega). The presented data is the calculated mean of three independent experiments where statistical analysis by *t*-test indicates significant differences in respect to control parental cells (* *p*<0.01). **B**. Transfected MCF7 cells were analysed for integrin alpha-5 (Itgα5) (left panel) and Pax-5 (right panel) transactivation potential through reporter gene assays (Dual luciferase/Promega) using Itgα5-luc and CD19-luc constructs respectively. Normalization of *Firefly* luciferase activity in relative light units (RLU) was performed using non-inducible *Renilla* luciferase and presented as the calculated mean of three independent experiments where statistical analysis was determined by *t*-test in respect to control parental cells (* *p*<0.01). **C**. Transfected MCF7 cells were also evaluated for the expression of epithelial tight junction protein zonula occludens-1 (ZO-1) by Western blot where GAPDH was used as a control.

We next set out to assess the ability of Pax-5 to modulate breast cancer cell adherence to fibronectin (FN), a component of ECM. Using the MCF7 epithelial-dominant cell line, we evaluated FN binding following *Pax-5* transfection (Figure 4A). We found that *Pax-5*-transfected cells increased cellular adherence to FN by 74% over vector-transfected control cells. In contrast, cellular adherence to bovine serum albumin (BSA), used as a control, remained unchanged in all cell conditions.

To further explore the molecular pathways leading to Pax-5-mediated enhanced adherent properties, we investigated the possible modulation of integrin-α5, a surface receptor specific for fibronectin, using dual luciferase-based reporter gene assays. Cells were co-transfected with *Pax-5* and the human integrin-alpha-5 promoter region cloned upstream from the firefly *luciferase* gene (Itgα5-luc) [29]. We found that *Pax-5* induced greater Itgα5-dependent luciferase activity (6.8 fold) over control cells (Figure 4B). To ascertain that recombinant *Pax-5* was transcriptionally active, we made use of another reporter construct bearing tandem repeats of Pax-5 binding motifs (CD19-luc) [30]. As expected, *Pax-5*-transfected cells revealed greater CD19-luciferase reporter activity (Figure 4B). Our observations suggest a role for *Pax-5* in Itgα5 upregulation. These findings also support the observed increase of cell adhesion from *Pax-5* bearing cells.

We next evaluated whether *Pax-5* could modulate the expression of tight junction protein ZO-1 (Zona occludens-1), an important regulator for mammary epithelial adhesion and cell junction integrity. Using Western blot, we found that *Pax-5* transfected MB231 cells led to greater protein expression levels of ZO-1 in comparison to vector-transfected control cells (Figure 4C). Altogether our data suggest that *Pax-5* promotes breast cancer cell adhesion and induces pro-adherent gene expression signatures.

### Pax-5 represses breast cancer aggressiveness

**Figure 5:**
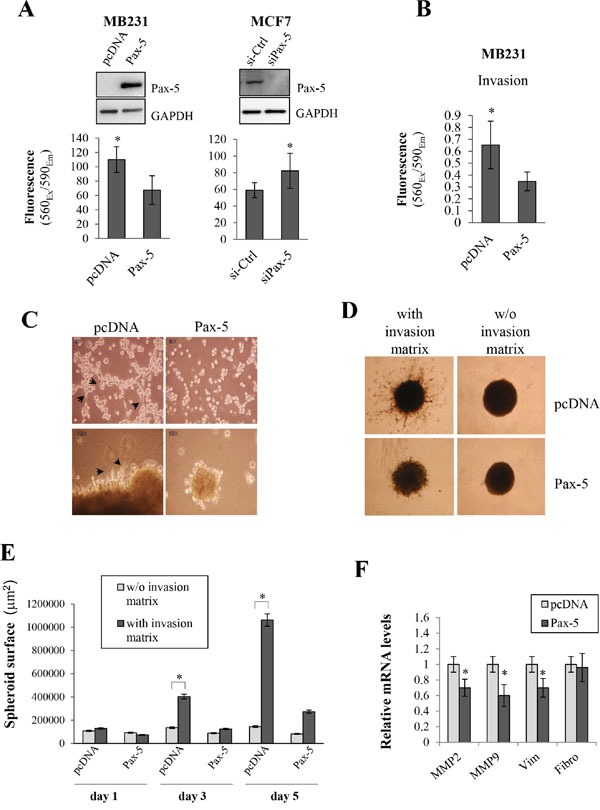
Pax-5 suppresses breast cancer cell malignant features **A**. Cell migration was monitored in MB231cells (left panel/transfected with pcDNA or Pax-5) and MCF7 cells (right panel/transfected with a Pax-5 silencing siRNA (siPax-5) or a control scrambled siRNA (si-Ctrl)). Pax-5 and GAPDH protein expression levels were also validated by Western blot (top panels) for each respective breast cancer models. **B**. MB231 cells transfected with either Pax-5 or pcDNA were monitored for cell invasion processes and **C**. anchorage-independent growth. Images were taken at a 40X (top panels) and 100X (bottom panels) magnifications where black arrows point to elongated mesenchymal-like cells. **D**. Stably transfected BT549 cells with Pax-5 were evaluated for 3D spheroid formation assays with or without (w/o) the presence of invasion matrix and **E**. quantified using the ImageJ software to assess the total surface invaded by migrating cells. **F**. Transfected MB231 cells were also monitored for matrix metallo-proteinases (MMP-2 and MMP-9), vimentin (Vim) and fibronectin (Fibro) using RT-qPCR. The presented data is the calculated mean of three independent experiments where statistical analysis was determined by *t*-test in respect to control cells (* *p*<0.01).

Given the epithelial-like characteristics induced by *Pax-5* in breast cancer cells, we wanted to assess the effects of *Pax-5* on cancer cell malignancy in terms of aggressive cancer cell processes (i.e. migration, invasion, anchorage-independent growth, and spheroid formation in 3D cultures). We first made use of the aggressive mesenchymal-dominant MB231 breast cancer model to study cellular migration following *Pax-5* transfection. Using chamber well inserts, we found that *Pax-5* decreased MB231 migration by 39% when compared to vector control cells (Figure 5A). To confirm our observations, we repeated the experiment using epithelial-dominant MCF7 cells which were transfected with either a siRNA targeting Pax-5 or, a scrambled non-targeting siRNA as a control. Loss of *Pax-5* expression led to an increase (near 40%) of MCF7 migration properties in comparison to non-targeting siRNA controls (Figure 5A). Western blots were also performed in parallel to verify the respective knocked-up and knocked-down expression models of Pax-5. We next evaluated breast cancer invasion and its modulation by *Pax-5*. Using matrigel-filled invasion chambers, we monitored invasion properties in *Pax-5* transfected MB231 cells. We found that *Pax-5* recombinant expression in MB231 cells significantly decreased invading cells by 48% (Figure 5B).

To further elucidate Pax-5 function in breast cancer processes, we performed various embedded culture condition to assess other breast cancer processes. First, we seeded MB231 cells on soft agar beds and evaluated their capacity for anchorage-independent growth following *Pax-5* transfection. As expected, control MB231 cells displayed morphological characteristics of mesenchymal-like features (i.e. elongated pods and filiform cell shapes) (Figure 5C). On the other hand, *Pax-5* transfected cells were more condensed with cubic cellular shapes reminiscent of epithelial-like cells. In addition, MB231 control cell colonies were greater in number and size when compared to *Pax-5* transfected cells (data not shown). To confirm our observations, we also performed 3D spheroid assays (with ECM) where the total surface of migrating cells was evaluated over time. We found that spheroids bearing *Pax-5* expression were not only smaller in size; but also, were devoid of migrating cells out from the center spheroid (Figure 5D). As expected, control samples without invasion matrix did not invade the surrounding area. We then plotted the total surface invaded by migrating cells and found that *Pax-5* significantly abrogates spheroid migration activity and matrix invasion over time (75% inhibition at day 5) (Figure 5E).

To expose some molecular mechanisms by which *Pax-5* could suppress breast cancer aggressive features, we performed RT-qPCR on molecular mediators of breast cancer malignancy such as: MMP2 and MMP9; vimentin and fibronectin following *Pax-5* transfection of MB231 cells. We found that *Pax-5* expression significantly decreased *MMP2* (30%), *MMP9* (39%) and *vimentin* (30%) expression when compared to MB231 cells transfected with the vector alone (Figure 5F). No changes were observed for fibronectin expression. Altogether, these results strongly suggest that *Pax-5* mitigates aggressive cellular and molecular processes which are essential for breast cancer disease progression.

### Pax-5 induces E-cadherin expression and MET in breast cancer cells

**Figure 6:**
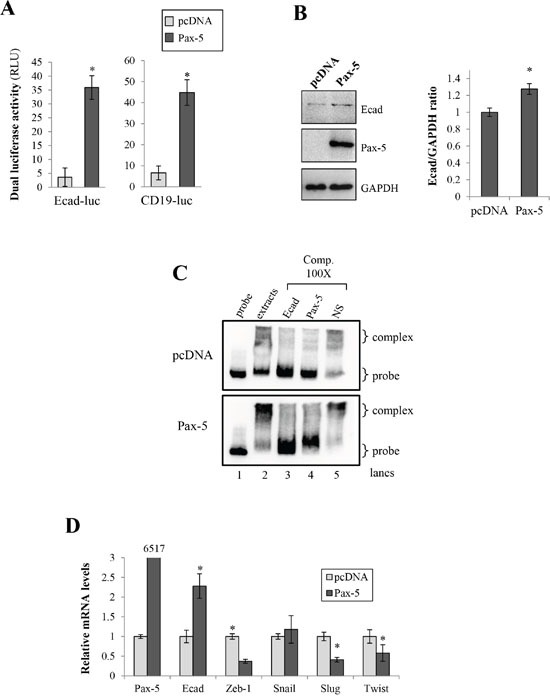
Pax-5 induces E-cadherin expression in breast cancer cells **A**. MB231 cells transfected with either the empty vector (pcDNA) or Pax-5 were analyzed for E-cadherin (left panel) and CD19 (right panel) promoter transactivation potential using dual reporter gene assays (Promega) with Ecad-luc and CD19-luc (Pax-5 responsive reporter) constructs respectively. Normalization of firefly luciferase activity was performed using non-inducible *Renilla* luciferase and plotted in relative light units (RLU) in relation to parental cells. **B**. E-cadherin was monitored in transfected cells by Western blot (left panel) where protein density was quantified and plotted using the ImageJ software plotted (right panel). **C**. Electrophoretic Mobility Shift Assays (EMSA) were conducted on nuclear extracts from Pax-5 transfected BT549 cells and mixed with biotin-labelled probes corresponding to a partial region of the E-cadherin promoter. DNA-protein complexes were resolved and analyzed on a 4% polyacrylamide gel. Specific (Ecad and Pax-5) and non-specific (NS) cold competition (Comp.) assays were carried out by adding a 100-fold molar excess of unlabeled dsDNA oligonucleotides corresponding to the E-cadherin (Ecad) or the Pax-5 consensus binding motif. Control lanes were loaded with free probe (probe) and extracts from empty vector transfected cells (pcDNA). **D**. Transfected cells were also monitored for Pax-5, E-cadherin, and E-cadherin suppressors (Zeb, Snail, Slug and Twist) using RT-qPCR. The presented data is the calculated mean of three independent experiments where statistical analysis was determined by *t*-test in respect to control cells (* *p*<0.01).

Our findings suggest that *Pax-5* promotes epithelial characteristics while concomitantly reducing mesenchymal features in breast cancer cells; a process reminiscent of MET. Previous reports have shown that E-cadherin downregulation is thought to be a primary contributor to the onset of EMT [3–5]. We thus investigated the possible modulation of E-cadherin transcriptional activity by *Pax-5* using reporter gene assays. MB231 cells were co-transfected with *Pax-5* and the promoter region from the E-cadherin cloned upstream from the *luciferase* gene (Ecad-luc). Impressively, a near 10-fold increase of Ecad-luc activity was observed in cells bearing *Pax-5* overexpression (Figure 6A). To support our findings, we performed Western blots on *Pax-5* transfected cells and observed a 27% increase of total E-cadherin protein expression when compared to control vector cells (Figure 6B). To evaluate whether E-cadherin could represent a potential target gene for the Pax-5 transcription factor, we studied the E-cadherin promoter region (-3kb) for Pax-5 binding motifs using the Promo software tool [31] and found multiple putative Pax-5 consensus binding motifs (data not shown). To validate the binding affinity of Pax-5 to these latter motifs, we conducted EMSA experiments using the E-cadherin promoter as a dsDNA probe. We found that nuclear extracts from Pax-5-transfected cells led to a shifted complex (lane 2) which was nearly absent in vector-transfected controls (Figure 6C). To validate the components of the shifted complex, we made use of specific competitions with 100-fold excess of non-labeled dsDNA sequences corresponding to the E-cadherin promoter region (lane 3), the Pax-5 consensus motif (lane 4) and an irrelevant non-specific sequence (lane 5). As expected, the addition of the cold E-cadherin promoter sequences outcompeted the visible shifted complex (lane 3) while the irrelevant non-specific dsDNA did not (lane 5). More interestingly, the addition of cold competition comprising of the Pax-5 motif also abrogated the shifted complexes (lane 4) demonstrating the presence of Pax-5 in the protein/DNA complex.

To determine whether Pax-5 regulates known E-cadherin repressors, we performed RT-qPCR on Zeb-1, Snail, Slug, and Twist in Pax-5 transfected MB231 cells. As expected, we observed an increase of both *Pax-5* and *E-cadherin* transcripts (Figure 6D) following *Pax-5* transfection. In contrast, *Pax-5* significantly inhibited the expression of *Zeb-1*, *Slug* and *Twist*. Altogether, our results demonstrate that Pax-5 is capable of binding the E-cadherin promoter and induce E-cadherin expression. In addition, *Pax-5* inhibits the expression of E-cadherin repressors.

**Figure 7:**
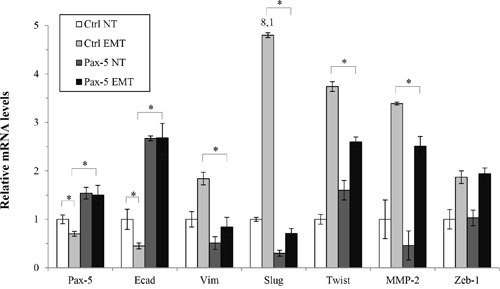
Pax-5 suppresses epithelial to mesenchymal phenotypic transitioning processes Transfected MCF7 cells with either the empty pcDNA (Ctrl) or Pax-5 were transiently induced for epithelial to mesenchymal transitioning (EMT) with TGF-β and TNF-α (10 ng/ml each) treatments for 48 hours. Cells were then monitored for EMT markers and E-cadherin regulators using RT-qPCR. The presented data is the calculated mean of three independent experiments where statistical analysis was determined by *t*-test in respect to control cells (* *p*<0.01).

Given that E-cadherin is a potent inhibitor of EMT, we set out to examine whether *Pax-5* could mitigate transiently-induced EMT in breast cancer cells. Accordingly, MCF7 were induced for EMT by TGFβ/TNFα treatments and examined for phenotypic marker expression by RT-qPCR. As expected, EMT induction of MCF7 cells led to an increase of mesenchymal gene expression such as: vimentin, Slug, Twist, MMP-2 and Zeb1 (Figure 7). Following EMT induction, we also observed a concomitant reduction (55%) of epithelial marker E-cadherin transcripts. Interestingly, we also detected a decrease of *Pax-5* expression (30%) suggesting the loss of *Pax-5* expression during breast cancer EMT. However, when we evaluated the phenotypic marker profiles from TGFβ/TNFα-induced EMT in *Pax-5* transfected cells, *Pax-5* suppressed the majority of phenotype markers associated to mesenchymal-like features (i.e. vimentin, Slug, Twist and MMP-2 expression). More importantly, *Pax-5* blocked the EMT-mediated decline of E-cadherin expression. Collectively, our results suggest that *Pax-5* not only sustains epithelialisation; but also, inhibits EMT.

## DISCUSSION

While the majority of research conducted on breast cancer metastasis has largely focused on factors initiating metastasis, the mechanisms governing EMT-MET during the metastatic cascade are relatively unknown. In this study, we provide evidence for the *Pax-5* gene to regulate breast cancer cell phenotype identity and EMT-MET processes. More interestingly, we found that in contrast to its oncogenic effects in lymphoid-derived cancers, Pax-5 confers a tumor suppressor role in breast carcinoma.

Our results provide the largest compilation of *Pax-5* expression profiles in mammary tissues and demonstrate that *Pax-5* is almost ubiquitously expressed (97%) in all breast cancer cell lines and clinical tissues examined. These results are in agreement with recently published data from breast cancer cell lines [18, 23, 24] and primary breast cancer tissues [25, 32]. Despite these observations, we were unable to correlate *Pax-5* signature patterns to specific mammary pathologies, tumor grade or staging (data not shown). This may be due to the origin and source of tissue samples tested (all consisting of primary tumor specimens) which do not represent *Pax-5* expression profiles in circulating tumor cells or metastatic niches.

In general, we observe that Pax-5 has an anti-tumorigenic role in breast cancer cells. These findings are consistent with previous observations in breast and liver carcinoma cell lines, where *Pax-5* overexpression led to cell cycle arrest and reduced growth rates [18, 26]. Mechanistically, we observed that *Pax-5* inhibits ERK activation pathways which may support *Pax-5*-induced attenuation of breast cancer growth. We also demonstrate that *Pax-5* suppresses cellular anchorage-independent growth, migration and invasion; processes all involved in breast cancer malignancy. Our observations corroborate those from Schebesta *et al*. (2007) who demonstrate that *Pax-5* leads to the suppression of migratory features with a concomitant increase of cell adhesion properties [17].

More interestingly, we found that *Pax-5* is a key player in breast cancer cell epithelialisation through the suppression of malignant features and inhibition of EMT. From a mechanistic standpoint, we found that *Pax-5* negatively regulates the expression of key mesenchymal regulators while concomitantly up-regulates epithelial markers reminiscent of MET. First, we observed that *Pax-5* induces the epithelial marker ZO-1 and most importantly E-cadherin. ZO-1 is a tight junction protein that is lost with the induction of the EMT [33, 34]. E-cadherin is also an epithelial cell surface marker in addition to a potent EMT-MET regulator. Structurally, surface E-cadherin binds catenins to the cell's membrane sequestering catenin shuttling to the nucleus and, avoiding the activation of mesenchymal genes [2, 10, 11]. Consequently, loss of E-cadherin not only disrupts cell–cell junctions; but also, induces cancer cell malignancy leading to tumor progression *in vitro* and *in vivo* [6, 7, 35]. Accordingly, we found that *Pax-5* modulates E-cadherin expression through both direct and indirect mechanisms. For instance, we found Pax-5 to bind and transactivate the human E-cadherin promoter region. These results are supported by ChIP-sequencing data provided by the ENCODE project (HudsonAlpha Institute for Biotechnology) demonstrating that E-cadherin promoter regions are co-immunoprecipitated by Pax-5 antibodies [36]. On the other hand, we demonstrate that *Pax-5* negatively regulates transcriptional repressors of E-cadherin such as Slug, Zeb and Twist. These findings strongly suggest that *Pax-5* is a potent modulator of E-cadherin expression and epithelial cell identity.

Our findings strongly support a beneficial role for Pax-5 expression in breast cancer tumors. These results concur with reports showing that elevated *Pax-5* expression in primary breast tumor sites correlates with lower risk of disease progression and relapse [16]. However, the role of Pax-5 in epithelial cell identity and EMT-MET processes may present dichotomous outcomes for breast cancer patients. For example, our results show that *Pax-5* opposes mesenchymal and invasive behavior reminiscent of MET; a process also required for successful colonization of secondary tumors during the metastatic cascade [1, 37, 38]. In other words, the phenotype transition triggered by *Pax-5* may foster MET in circulating cancer cells enabling the establishment of distant metastatic niches. Accordingly, several lines of evidence suggest a role for *Pax-5* in the metastatic cascade and disease progression. For example, some researchers demonstrated that *Pax-5* expression levels correlate with more aggressive forms of astrocytomas [39], neuroendocrine tumors [40], and pulmonary carcinomas [41]. Others have also shown that the presence of *Pax-5* in transitional cell carcinomas of the bladder is associated with a fourfold relative risk of malignancy [42]. Another study by Ellsworth *et al*. (2009) revealed that *Pax-5* expression levels are 100-fold higher in microdissected breast cancer cells found in lymph nodes in comparison to their primary tumor counterparts [25]. These observations coincide with a role for *Pax-5* to orchestrate the attenuation of mesenchymal properties and reboot epithelial characteristics during the metastatic cascade.

The seemingly conflicting functions exhibited by *Pax-5* on breast cancer malignancy and progression appear to be highly dependent on particular cellular contexts during the metastatic cascade. We hypothesize that triggered expression of *Pax-5* in circulating cancer cells would induce MET and concomitant suppression of mesenchymal gene expression. As a result, Pax-5 would enhance adherent features allowing cells to colonize and establish secondary tumors. However, it is important to note that the effects of *Pax-5* on cancer progression would be time and context-dependent and that *Pax-5* up-regulation during the initial (primary tumor) or later events could result in different pathological outcomes.

In summary, this study brings new insight in the molecular biology of breast carcinogenesis. Our results support a role for *Pax-5* as a potent regulator of cell signaling events leading to the development, maintenance and progression of breast cancer disease. The prevalence of *Pax-5* expression in mammary tissues in addition to its involvement in malignant and phenotypic transitioning processes, presents *Pax-5* as a potential biomarker for breast cancer tissues. We thus believe that further studies will better define and elucidate *Pax-5*-mediated pathways, and advance our discovery of more effective treatments and diagnostics strategies for breast cancer patients.

## EXPERIMENTAL PROCEDURES

### Cells lines, tissues and treatments

The HEK293 (CRL-1573); MCF7 (HTB-22); T47D (HTB-133); MCF12A (CRL-10782); MCF10A (CRL-10317); MDA-MB231 (HTB-26); MDA-MB415 (HTB-128); MDA-MB436 (HTB-130); MDA-MB468 (HTB-132); BT549 (HTB-122); BT474 (HTB-20); and HCC 1954 (CRL-2338) cell lines were obtained from the American Type Culture Collection (Rockville, MD, USA). The REH and the Nalm-6 cell lines were kindly provided by Dr. Edward A. Clark (University of Washington Medical Center, Seattle). Tissue microarrays, represented in duplicate (306 core samples total), were purchased from US Biomax Inc. (Rockville, MD, USA). We made use of arrays T082, BRC961, BR961 and BR1006 which contained a variety of healthy mammary tissues, benign and malignant tumors (Supplementary Table 2).

MCF7 cells were maintained in complete media (10% fetal bovine serum (FBS) (Hyclone Laboratories, Logan, UT, USA) L-glutamine (2 mM), penicillin (100 units/mL), and streptomycin (100 μg/mL) (Invitrogen/Life Technologies, Burlington, Canada)) with DMEM low glucose medium. B-cells and HCC1954 were maintained in complete media with RPMI 1640. HEK293, MDA-MB231, MDA-MB415, MDA-MB436, MDA-MB468 and BT474 cell lines were cultured with complete media in DMEM high glucose medium. T47D and BT549 were cultured with complete media in RPMI 1640 medium supplemented with bovine insulin (0.01 mg/mL). MCF12A and MCF10A were maintained with complete media in DMEM/F12 medium supplemented with sodium pyruvate (1 mM), bovine insulin (10 μg/mL), EGF (20 ng/mL), cholera toxin (100 ng/mL), and hydrocortisone (500 ng/mL). We also made use of transforming growth factor-beta (TGF-β) and tumor necrosis factor-alpha (TNF-α) treatments (10 ng/ml each) for 48 h to induce transient EMT [43].

### Plasmids and transfections

The *Pax-5* recombinant gene was cloned as described previously [44]. We also made use of *firefly*-luciferase reporter constructs E-cadherin-luc [45]; integrin-alpha5-luc [29]; and, CD19-luc [30]. Transfections of DNA plasmids were performed as previously described [46] with the XtremeGene 9 reagent (Roche, Branford, CT, USA). Gene suppression experiments were accomplished using a transfection mixture of siRNA targeting Pax-5 (ON-TARGETplus, Dharmacon, Rockford, IL, USA). Cells were seeded in six-well plates (5×10^5^ cells/well), grown for 24 h, and transfected with 100 pmol of siRNA using Lipofectamine 2000 (Invitrogen). Control cells were treated with a non-specific (non-silencing) siRNA (Dharmacon).

### PCR and quantitative real-time RT-PCR

Reverse transcriptions were performed on purified total RNA retrieved either from live cells by Trizol Reagent (Invitrogen) or from paraffin-embedded tissues by RNA isolation kits provided by Arcturus® Paradise® Plus (Molecular Device, Downingtown, PA, USA) according to manufacturer's instructions. Levels of gene expression were verified by quantitative real-time RT-PCR (RT-qPCR) as previously described [46] using specific PCR primer pairs (Supplementary Table 3). Comparative expression levels were calculated using the ΔΔCt method of Livak and Schmittgen, (2001) [47], using the hypoxanthine ribosyltransferase (HPRT) transcript as a normalizing control.

### Immunohistological analysis

Immunohistochemistry was performed on formalin-fixed paraffin-embedded (FFPE) breast samples using commercial tissue arrays (US Biomax Inc.) Samples were deparaffinized by baking the slides at 60°C for 30 min and rehydrated with ethanol and TBS-T (0.05%). Following a heat-induced epitope retrieval method, samples were immersed in a boiling solution of 1 mM EDTA, 0.05% Tween, pH 8.0 for 20 min and rinsed in TBS-T. Antibody staining was then conducted using the UltraVision ONE Detection System (Lab Vision, Thermo Scientific, Ottawa, Canada) according to manufacturer's protocol. Relative protein expression was performed blinded using a standardized grid imposed on images captured of at least two random stained tissue sections.

### Cell proliferation and apoptosis assays

Cellular viability was monitored using the CellTiter-Blue (Promega, Madison, WI, USA) assay as described previously [46]. Briefly, cells were seeded in 96 well plates (2×10^3^ cells/well) with the indicated conditions where 20 μL of CellTiter-Blue substrate was added up to 100 μL/well and incubated for 1 hour at 37°C. Thereafter, microplates were subjected to a fluorescence analysis on a plate reader (560_Ex_/590_Em_). From here, apoptotic events could also be measured on the same microplate by the addition of 120 μL of Apo-ONE (Promega), an additional incubation (1 hour) and analysis by fluorescence (485_Ex_/520_Em_).

### Western blot analysis

Western blots were performed as previously described [46] in whole cell lysate (WCL) buffer. The membrane was blocked with 5% milk for 1 hour and incubated thereafter with various antibodies against: Pax-5 (exon 5/6, New England Peptide, Gardner, MA, USA); Pax-5 (A-11, Santa Cruz Biotechnology); Actin (Sigma); GAPDH (Cell Signaling Technologies, Danvers, MA, USA); ERK 1/2 and phosphorylated-ERK1/2 (Cell Signaling Technologies); AKT and phosphorylated-AKT (Cell Signaling Technologies); ZO-1 (Invitrogen); and E-cadherin (BD Biosciences, Mississauga, ON, Canada). Densitometry calculations were performed (where indicated) with the Image Processing and Analysis in Java (ImageJ) program (http://rsbweb.nih.gov/ij/) where protein expression levels were normalized using a housekeeping control.

### Cell migration and invasion assays

Migration and invasion assays were performed as previously described [46]. Only for the invasion assays, Matrigel (Corning Life Sciences, Tewksbury MA, USA) was added (50 μL) to the well inserts and incubated overnight. Cells (5×10^4^) were then seeded into transwell inserts with (invasion) or without Matrigel (migration) in serum-free media whereas the bottom receiver plates received complete DMEM media. Plates were incubated at 37°C for 24 h where the invaded cells were quantified by CellTiter-Blue.

### Cell adhesion and 3D cultures

To monitor cell adhesion to extracellular matrix component fibronectin, cells (2×10^4^ cells/well) were added to 96 well plates pre-coated with fibronectin or serum albumin (BioCoat, BD Biosciences) and incubated for 45 minutes at 37°C in a humidified incubator containing 5% CO_2_. Unbound cells were gently washed out from the wells leaving adherent cells which were quantified using the CellTiter-Blue method. Anchorage-independent growth was monitored in 6-well plates containing 0.3% top low-melt agarose-0.8% bottom low-melt agarose (Difco, BD Biosciences). Cell colonies were then photographed and analysed. 3D-spheroid cultures were performed according to the manufacturer's instructions (Trevigen, Gaithersburg, MD, USA). Briefly, cells (3×10^3^ cells/well) were suspended in spheroid extracellular matrix and plated into 96 U-shaped well plates. After 3 days, invasion matrix containing chemoattractants was added to formed spheroids. Pictures and quantifications were then performed using the ImageJ software.

### Reporter gene assays

Luciferase-based reporter gene assays were conducted as described previously [44] using the Dual-Glo luciferase system (Promega). 24 h post-transfection, cells were lysed and analyzed for luciferase activity using a luminometer (BMG Fluostar, Fisher Scientific, Ottawa, Canada). Relative reporter activity was calculated and normalized based on *renilla*-luciferase activity which reflected transfection efficiency.

### Preparation of nuclear extracts and Electrophoretic Mobility Shift Assays (EMSA)

Nuclear extracts were prepared and analyzed by gel shift assays as previously described [44]. EMSAs were performed using the Gelshift™ Chemiluminescent kit from Active Motif (Carlsbad, CA USA) according to the manufacturer's instructions. Biotin-labelled oligonucleotides were synthesized (Supplementary Table 3) for the development of a labelled probe corresponding to the human E-cadherin promoter region by PCR on the E-cadherin reporter luciferase construct [45]. Cold competition assays were carried out by adding a 100-fold molar excess of unlabeled dsDNA oligonucleotide corresponding to: the E-cadherin promoter; the *Pax-5* motif; and a non-specific scrambled control.

## SUPPLEMENTARY MATERIALS TABLES



## References

[1] Chao YL, Shepard CR, Wells A (2010). Breast carcinoma cells re-express E-cadherin during mesenchymal to epithelial reverting transition. Mol Cancer.

[2] Wells A, Yates C, Shepard CR (2008). E-cadherin as an indicator of mesenchymal to epithelial reverting transitions during the metastatic seeding of disseminated carcinomas. Clin Exp Metastasis.

[3] Yao D, Dai C, Peng S (2011). Mechanism of mesenchymal-epithelial transition and the relationship with metastatic tumor formation. Mol Cancer Res.

[4] Vleminckx K, Vakaet L, Mareel M, Fiers W, van Roy F (1991). Genetic manipulation of E-cadherin expression by epithelial tumor cells reveals an invasion suppressor role. Cell.

[5] Berx G, Van Roy F (2001). The E-cadherin/catenin complex: an important gatekeeper in breast cancer tumorigenesis and malignant progression. Breast Cancer Res.

[6] Onder TT, Gupta PB, Mani SA, Yang J, Lander ES, Weinberg RA (2008). Loss of E-cadherin promotes metastasis via multiple downstream transcriptional pathways. Cancer Res.

[7] Perl AK, Wilgenbus P, Dahl U, Semb H, Christofori G (1998). A causal role for E-cadherin in the transition from adenoma to carcinoma. Nature.

[8] Hunt NC, Douglas-Jones AG, Jasani B, Morgan JM, Pignatelli M (1997). Loss of E-cadherin expression associated with lymph node metastases in small breast carcinomas. Virchows Arch.

[9] Siitonen SM, Kononen JT, Helin HJ, Rantala IS, Holli KA, Isola JJ (1996). Reduced E-cadherin expression is associated with invasiveness and unfavorable prognosis in breast cancer. Am J Clin Pathol.

[10] Baranwal S, Alahari SK (2009). Molecular mechanisms controlling E-cadherin expression in breast cancer. Biochem Biophys Res Commun.

[11] Conacci-Sorrell M, Simcha I, Ben-Yedidia T, Blechman J, Savagner P, Ben-Ze'ev A (2003). Autoregulation of E-cadherin expression by cadherin-cadherin interactions: the roles of beta-catenin signaling, Slug, and MAPK. J Cell Biol.

[12] Cano A, Perez-Moreno MA, Rodrigo I, Locascio A, Blanco MJ, MG del Barrio, Portillo F, Nieto MA (2000). The transcription factor snail controls epithelial-mesenchymal transitions by repressing E-cadherin expression. Nat Cell Biol.

[13] Yang J, Mani SA, Donaher JL, Ramaswamy S, Itzykson RA, Come C, Savagner P, Gitelman I, Richardson A, Weinberg RA (2004). Twist, a master regulator of morphogenesis, plays an essential role in tumor metastasis. Cell.

[14] Hajra KM, Chen DY, Fearon ER (2002). The SLUG zinc-finger protein represses E-cadherin in breast cancer. Cancer Res.

[15] Eger A, Aigner K, Sonderegger S, Dampier B, Oehler S, Schreiber M, Berx G, Cano A, Beug H, Foisner R (2005). DeltaEF1 is a transcriptional repressor of E-cadherin and regulates epithelial plasticity in breast cancer cells. Oncogene.

[16] Crapoulet N, O'Brien P, Ouellette RJ, Robichaud GA (2011). Coordinated Expression of Pax-5 and FAK1 in Metastasis. Anticancer Agents Med Chem.

[17] Schebesta A, McManus S, Salvagiotto G, Delogu A, Busslinger GA, Busslinger M (2007). Transcription factor Pax5 activates the chromatin of key genes involved in B cell signaling, adhesion, migration, and immune function. Immunity.

[18] Vidal LJ, Perry JK, Vouyovitch CM, Pandey V, Brunet-Dunand SE, Mertani HC, Liu DX, Lobie PE (2010). PAX5alpha enhances the epithelial behavior of human mammary carcinoma cells. Mol Cancer Res.

[19] Strachan T, Read AP (1994). PAX genes. Current Opinion in Genetics & Development.

[20] Busslinger M (2004). Transcriptional control of early B cell development. Annu Rev Immunol.

[21] Hamada T, Yonetani N, Ueda C, Maesako Y, Akasaka H, Akasaka T, Ohno H, Kawakami K, Amakawa R, Okuma M (1998). Expression of the PAX5/BSAP transcription factor in haematological tumour cells and further molecular characterization of the t(9;14)(p13;q32) translocation in B-cell non-Hodgkin's lymphoma. British Journal of Haematology.

[22] O'Brien P, Morin P, Ouellette RJ, Robichaud GA (2011). The Pax-5 gene: a pluripotent regulator of B-cell differentiation and cancer disease. Cancer Res.

[23] Vouyovitch CM, Vidal L, Borges S, Raccurt M, Arnould C, Chiesa J, Lobie PE, Lachuer J, Mertani HC (2008). Proteomic analysis of autocrine/paracrine effects of human growth hormone in human mammary carcinoma cells. Adv Exp Med Biol.

[24] Palmisano WA, Crume KP, Grimes MJ, Winters SA, Toyota M, Esteller M, Joste N, Baylin SB, Belinsky SA (2003). Aberrant promoter methylation of the transcription factor genes PAX5 alpha and beta in human cancers. Cancer Res.

[25] Ellsworth RE, Seebach J, Field LA, Heckman C, Kane J, Hooke JA, Love B, Shriver CD (2009). A gene expression signature that defines breast cancer metastases. Clin Exp Metastasis.

[26] Liu W, Li X, Chu ES, Go MY, Xu L, Zhao G, Li L, Dai N, Si J, Tao Q, Sung JJ, Yu J (2011). Paired box gene 5 is a novel tumor suppressor in hepatocellular carcinoma through interaction with p53 signaling pathway. Hepatology.

[27] Mhawech-Fauceglia P, Saxena R, Zhang S, Terracciano L, Sauter G, Chadhuri A, Herrmann FR, Penetrante R (2007). Pax-5 immunoexpression in various types of benign and malignant tumours: a high-throughput tissue microarray analysis. Journal of Clinical Pathology.

[28] Neve RM, Chin K, Fridlyand J, Yeh J, Baehner FL, Fevr T, Clark L, Bayani N, Coppe JP, Tong F, Speed T, Spellman PT, DeVries S, Lapuk A, Wang NJ, Kuo WL (2006). A collection of breast cancer cell lines for the study of functionally distinct cancer subtypes. Cancer Cell.

[29] Qin L, Chen X, Wu Y, Feng Z, He T, Wang L, Liao L, Xu J (2011). Steroid receptor coactivator-1 upregulates integrin alpha(5) expression to promote breast cancer cell adhesion and migration. Cancer Res.

[30] Nutt SL, Morrison AM, Dorfler P, Rolink A, Busslinger M (1998). Identification of BSAP (Pax-5) target genes in early B-cell development by loss- and gain-of-function experiments. EMBO J.

[31] Messeguer X, Escudero R, Farre D, Nunez O, Martinez J, Alba MM (2002). PROMO: detection of known transcription regulatory elements using species-tailored searches. Bioinformatics.

[32] Edgar R, Domrachev M, Lash AE (2002). Gene Expression Omnibus: NCBI gene expression and hybridization array data repository. Nucleic Acids Research.

[33] Lee H, O'Meara SJ, O'Brien C, Kane R (2007). The role of gremlin, a BMP antagonist, and epithelial-to-mesenchymal transition in proliferative vitreoretinopathy. Invest Ophthalmol Vis Sci.

[34] Reichert M, Muller T, Hunziker W (2000). The PDZ domains of zonula occludens-1 induce an epithelial to mesenchymal transition of Madin-Darby canine kidney I cells. Evidence for a role of beta-catenin/Tcf/Lef signaling. J Biol Chem.

[35] Lehembre F, Yilmaz M, Wicki A, Schomber T, Strittmatter K, Ziegler D, Kren A, Went P, Derksen PW, Berns A, Jonkers J, Christofori G (2008). NCAM-induced focal adhesion assembly: a functional switch upon loss of E-cadherin. EMBO J.

[36] Birney E, Stamatoyannopoulos JA, Dutta A, Guigo R, Gingeras TR, Margulies EH, Weng Z, Snyder M, Dermitzakis ET, Thurman RE, Kuehn MS, Taylor CM, Neph S, Koch CM, Asthana S, Malhotra A (2007). Identification and analysis of functional elements in 1% of the human genome by the ENCODE pilot project. Nature.

[37] Chaffer CL, Brennan JP, Slavin JL, Blick T, Thompson EW, Williams ED (2006). Mesenchymal-to-epithelial transition facilitates bladder cancer metastasis: role of fibroblast growth factor receptor-2. Cancer Res.

[38] Hugo H, Ackland ML, Blick T, Lawrence MG, Clements JA, Williams ED, Thompson EW (2007). Epithelial--mesenchymal and mesenchymal--epithelial transitions in carcinoma progression. J Cell Physiol.

[39] Stuart ET, Kioussi C, Aguzzi A, Gruss P (1995). PAX5 expression correlates with increasing malignancy in human astrocytomas. Clin Cancer Res.

[40] Dong HY, Liu W, Cohen P, Mahle CE, Zhang W (2005). B-cell specific activation protein encoded by the PAX-5 gene is commonly expressed in merkel cell carcinoma and small cell carcinomas. The American Journal of Surgical Pathology.

[41] Kanteti R, Nallasura V, Loganathan S, Tretiakova M, Kroll T, Krishnaswamy S, Faoro L, Cagle P, Husain AN, Vokes EE, Lang D, Salgia R (2009). PAX5 is expressed in small-cell lung cancer and positively regulates c-Met transcription. Lab Invest.

[42] Adshead JM, Ogden CW, Penny MA, Stuart ET, Kessling AM (1999). The expression of PAX5 in human transitional cell carcinoma of the bladder: relationship with de-differentiation. BJU Int.

[43] Guan F, Handa K, Hakomori SI (2009). Specific glycosphingolipids mediate epithelial-to-mesenchymal transition of human and mouse epithelial cell lines. Proc Natl Acad Sci U S A.

[44] Robichaud GA, Nardini M, Laflamme M, Cuperlovic-Culf M, Ouellette RJ (2004). Human Pax-5 C-terminal isoforms possess distinct transactivation properties and are differentially modulated in normal and malignant B cells. J Biol Chem.

[45] Liu YN, Lee WW, Wang CY, Chao TH, Chen Y, Chen JH (2005). Regulatory mechanisms controlling human E-cadherin gene expression. Oncogene.

[46] Picot N, Guerrette R, Beauregard AP, Jean S, Michaud P, Harquail J, Benzina S, Robichaud GA (2015). Mammaglobin 1 promotes breast cancer malignancy and confers sensitivity to anticancer drugs. Mol Carcinog.

[47] Livak KJ, Schmittgen TD (2001). Analysis of relative gene expression data using real-time quantitative PCR and the 2(-Delta Delta C(T)) Method. Methods.

