# Supplementary material for: Pax-5 is a potent regulator of E-cadherin and breast cancer malignant processes
Source: Oncotarget. 2017 Jan 5;8(7):12052–66. doi: 10.18632/oncotarget.14511 (PMC5355325; doi:10.18632/oncotarget.14511)
Supplement: Supplementary file 1 [file oncotarget-08-12052-s001.pdf]

# Pax-5 is a potent regulator of E-cadherin and breast cancer malignant processes

## SUPPLEMENTARY TABLES

**Supplementary Table 1: Cell line models**

| Cells | Source | Pathology | Breast gene profile | Breast subtype |
|---|---|---|---|---|
| HEK293 | human, fetal embryonic kidney | non-cancerous embryonic kidney | N/A | N/A |
| REH | human lymphocyte | acute lymphocytic leukemia (non-T; non-B) | N/A | N/A |
| Nalm-6 | human lymphocyte | leukemia | N/A | N/A |
| MB415 | human breast; derived from pleural effusion | adenocarcinoma | ER+/PR- | luminal |
| MB436 | human breast; derived from pleural effusion | invasive ductal carcinoma | ER-/PR- | basal B |
| MB468 | human breast; derived from pleural effusion | adenocarcinoma | ER-/PR- | basal A |
| BT474 | human primary breast | invasive ductal carcinoma | ER+/PR+/ HER2 | luminal |
| BT549 | human primary breast | invasive ductal carcinoma | ER-/PR- | basal B |
| HCC1954 | human primary breast | ductal carcinoma | ER-/PR-/ HER2 | basal A |
| MCF7 | human breast; derived from pleural effusion | invasive ductal carcinoma | ER+/PR+ | luminal |
| MB231 | human breast; derived from pleural effusion | adenocarcinoma | ER-/PR- | basal B |
| T47D | human breast; derived from pleural effusion | invasive ductal carcinoma | ER+/PR+ | luminal |
| MCF12A | human primary breast | non-cancerous from fibrocystic disease | ER-/PR- | basal B |
| MCF10A | human primary breast | non-cancerous from fibrocystic disease | ER-/PR- | basal B |

Abbreviations: ER, estrogen receptor; PR, progesterone receptor; HER2, human epidermal growth factor receptor 2; ER/PR positivity, HER2 overexpression status are indicated. Cell line and expression data are derived from Neve et al. 2006. [28]

**Supplementary Table 2: Pax-5 protein expression in FFPE tissue arrays**

| Type/Pathology * | | Number (%) of Pax-5 expressing breast tissues and Scoring ** | | | | Total *** |
|---|---|---|---|---|---|---|
| | | Score 0 | Score 1 | Score 2 | Score 3 | |
| Normal Breast Tissue | | 1 (7%) | 1 (7%) | 9 (64%) | 3 (21%) | 14 |
| Cancer adjacent normal breast tissue | | 3 (27%) | 4 (36%) | 1 (9%) | 3 (27%) | 11 |
| Hyperplasia | | - | 5 (25%) | 11 (55%) | 4 (20%) | 20 |
| Benign (fibroadenosis, fibroadenoma) | | - | 9 (28%) | 18 (56%) | 5 (16%) | 32 |
| **Malignant** | Apocrine carcinoma + Carcinosarcoma | - | 4 (67%) | 2 (33%) | - | 6 |
| | Cysto/sarcoma phyllodes | - | 1 (6%) | 15 (83%) | 2 (11%) | 18 |
| | Invasive ductal carcinoma | - | 29 (23%) | 80 (63%) | 18 (14%) | 127 |
| | Invasive lobular carcinoma | - | 2 (25%) | 2 (25%) | 4 (50%) | 8 |
| | Medullary carcinoma | - | 3 (14%) | 10 (45%) | 9 (41%) | 22 |
| | Mixed lobular and ductal carcinoma | - | - | 2 (100%) | - | 2 |
| | Mucinous adenocarcinoma | 3 (14%) | 7 (32%) | 12 (55%) | - | 22 |
| | Neuroendocrine carcinoma | - | - | 2 (100%) | - | 2 |
| | Tubular carcinoma | 1 (17%) | 1 (17%) | 4 (67%) | | 6 |
| | Paget's disease | - | 2 (25%) | 5 (63%) | 1 (13%) | 8 |
| | DCIS + Intraductal carcinoma | - | 2 (25%) | 6 (75%) | - | 8 |
| | **Total** | 8 (3%) | 70 (22%) | 179 (58%) | 49 (16%) | 306 (100%) |

* As diagnosed by pathologists at Tissue Array Networks; DCIS = Ductal Carcinoma *In Situ*
** Score 0 = No expression, Score 1 = weak, Score 2 = moderate, Score 3 = strong
*** Total number of duplicate samples from T082, BRC961, BR961 and BR1006 TMAs
Abbreviation: DCIS = Ductal Carcinoma *in situ*

**Supplementary Table 3: PCR primers**

| | Forward primers (5'→3') | Reverse primers (5'→3') |
|---|---|---|
| **HPRT** | TGA CAC TGG CAA AAC AAT GCA | GGT CTT TTT CAC CGA CAA GCT |
| **Pax-5α** | GCG CAA GAG AGA CGA AGG T | CTG CTG CTG TGT GAA CAA GTC |
| **Pax-5β** | TTG GCA CGA GGT AGA CAC | AGC CCT CCA ACA CTG AGA |
| **Pax-5 (EMSA)** | Biotin-GAA TGG GGC ACT GAG GCG TG | Biotin-CAC GCC TCA GTG CCC CAT TC |
| **Ecad (EMSA)** | Biotin-CTT CAG CCC AGG AGT TC | Biotin-CGG TGG CTC ACT AAG AC |
| **CD19** | CCA CCT GGA GAT CAC TG | ATA AGC CAA AGT CAC AGC |
| **MMP2** | GCG GCG GTC ACA GCT ACT | GAC GCT CTT GAG ACT TTG |
| **MMP9** | GTG CTG GGC TGC TGC TTT GCT G | TTT CGA CTC TCC ACG GAT CTC T |
| **Vimentin** | CAA CCT GGC CGA GGA CAT | ACG CAT TGT CAA CAT CCT GTC T |
| **Fibronectine** | CCG CCG AAT GTA GGA CAA GA | TGC CAA CAG GAT GAC ATG AAA |
| **E-cadherin** | CAA GCT ATC CTT GCA CCT CAG | GCA TCA AGA GAA CTC CTA TCT TG |
| **Zeb-1** | GCC AAT AAG CAA ACG ATT CTG | TTT GGC TGG ATC ACT TTC AAG |
| **Snail** | GCT GCA GGA CTC TAA TCC AGA GTT | GAC AGA GTC CCA GAT GAG CAT TG |
| **Slug** | GCA GTG AGG GCA AGA AAA AG | GCG ATG CCC AGT CTA GAA AA |
| **Twist** | GGA GTC CGC AGT CTT ACG AG | TCT GGA CCT GGT AGA GG |
